# Skeletal muscles and gut microbiota-derived metabolites: novel modulators of adipocyte thermogenesis

**DOI:** 10.3389/fendo.2023.1265175

**Published:** 2023-10-06

**Authors:** Yi Tang, Ya-Di Wang, Yuan-Yuan Wang, Zhe-Zhen Liao, Xin-Hua Xiao

**Affiliations:** ^1^ Department of Metabolism and Endocrinology, The First Affiliated Hospital, Hengyang Medical School, University of South China, Hengyang, Hunan, China; ^2^ Department of Clinical Laboratory Medicine, The First Affiliated Hospital, Hengyang Medical School, University of South China, Hengyang, Hunan, China

**Keywords:** metabolite, signaling molecules, energy expenditure, obesity, adipocyte, skeletal muscles, gut microbiota

## Abstract

Obesity occurs when overall energy intake surpasses energy expenditure. White adipose tissue is an energy storage site, whereas brown and beige adipose tissues catabolize stored energy to generate heat, which protects against obesity and obesity-associated metabolic disorders. Metabolites are substrates in metabolic reactions that act as signaling molecules, mediating communication between metabolic sites (i.e., adipose tissue, skeletal muscle, and gut microbiota). Although the effects of metabolites from peripheral organs on adipose tissue have been extensively studied, their role in regulating adipocyte thermogenesis requires further investigation. Skeletal muscles and intestinal microorganisms are important metabolic sites in the body, and their metabolites play an important role in obesity. In this review, we consolidated the latest research on skeletal muscles and gut microbiota-derived metabolites that potentially promote adipocyte thermogenesis. Skeletal muscles can release lactate, kynurenic acid, inosine, and β-aminoisobutyric acid, whereas the gut secretes bile acids, butyrate, succinate, cinnabarinic acid, urolithin A, and asparagine. These metabolites function as signaling molecules by interacting with membrane receptors or controlling intracellular enzyme activity. The mechanisms underlying the reciprocal exchange of metabolites between the adipose tissue and other metabolic organs will be a focal point in future studies on obesity. Furthermore, understanding how metabolites regulate adipocyte thermogenesis will provide a basis for establishing new therapeutic targets for obesity.

## 1 Introduction

According to the World Obesity Atlas 2023 published by the World Obesity Federation, by 2035, more than half of the global population, exceeding 4 billion people, will be overweight or obese (1). Factors that have disrupted the balance between energy intake and expenditure over the last few decades include widespread availability and consumption of high-calorie palatable foods, the shift from active to sedentary lifestyles, and the prevalence of sleep deprivation (2, 3). These factors and medical technology innovations may have expedited the obesity epidemic. Obesity increases the risk of many metabolic dysfunctions and comorbidities, such as type 2 diabetes mellitus, cardiovascular disease, and cancer (4–6).

Mammals have three types of adipose tissues: white adipose tissue (WAT), brown adipose tissue (BAT), and beige adipose tissue. WAT stores energy (7), whereas BAT acts as a heat generator that maintains the core body temperature through the action of the mitochondrial protein, uncoupling protein 1 (UCP1) (8). Beige adipose tissues are a newly discovered class of fats that exhibit the qualities of white fats at rest and have browning potential upon activation by cold exposure or β3-adrenergic receptor agonists, promoting thermogenesis and energy expenditure (EE), improving the glucolipid metabolism, and showing great plasticity (9). In recent years, subcutaneous white adipocytes are converted into milk-producing glands formed by lipid-rich elements, referred to as pink adipocytes during pregnancy (10). Brown and beige adipocytes are thermogenic adipocytes that contain many dense mitochondria to dissipate energy in the form of heat (11). Activating BAT and inducing the browning of WAT can regulate systemic energy homeostasis, glucose and lipid metabolism, and insulin sensitivity (12). Hence, the modulation of the quantity and function of brown/beige adipocytes is a strategy to control human energy metabolism, providing a potential basis for the development of methods to treat obesity and other metabolic diseases (13).

Obesity is a complex chronic disease and its management requires a comprehensive approach. Four primary treatments for obesity include lifestyle changes (i.e., diet and exercise), cognitive-behavioral therapy, pharmacotherapy, and bariatric surgery (14). While medication and bariatric surgery are recommended for severe obesity, diet, exercise, and cognitive behavioral therapy are the primary strategies for the long-term management of obesity (15, 16). Kheniser et al. opined that a two-year lifestyle intervention results in a 5% reduction in weight; however, diet and exercise interventions also significantly improve obesity-related comorbidities and promote the remodeling of adipose tissue despite weight regain (16). Some organs secrete small molecules in response to modified dietary strategies and exercise, which are the factors contributing to the browning of WAT. These factors are considered potential therapeutic approaches for the treatment of obesity and related metabolic dysfunctions (17). One possible reason for the effectiveness of dietary strategies may be the alteration of functional metabolites in the gut microbiome, leading to the remission of obesity. Additionally, one factor that could influence the metabolic benefits of exercise is the secretion of myokines skeletal muscle (18, 19). Skeletal muscle and the gut microbiota are important contributors to endocrine function in the body and are involved in the development of various human diseases, including obesity and metabolic syndrome (20, 21). They communicate with other organs by secreting cytokines, exosomes, and metabolites, of which metabolites of which metabolites have gained significant attention recently as a popular research topic. Metabolites are often considered “fuel” or components of metabolic pathways (22). However, they also act as signaling molecules that mediate communication between metabolic organs (23). Intracellular metabolites regulate enzymatic activity and bind to nuclear receptors (24, 25). Extracellular metabolites also function by binding to membrane receptors (26). Studies have demonstrated that metabolites from skeletal muscle and the gut microbiota play important roles in systemic EE, such as influencing thermogenesis and adipose tissue browning (27, 28), rendering them suitable therapeutic targets for metabolic diseases.

**Figure 1:**
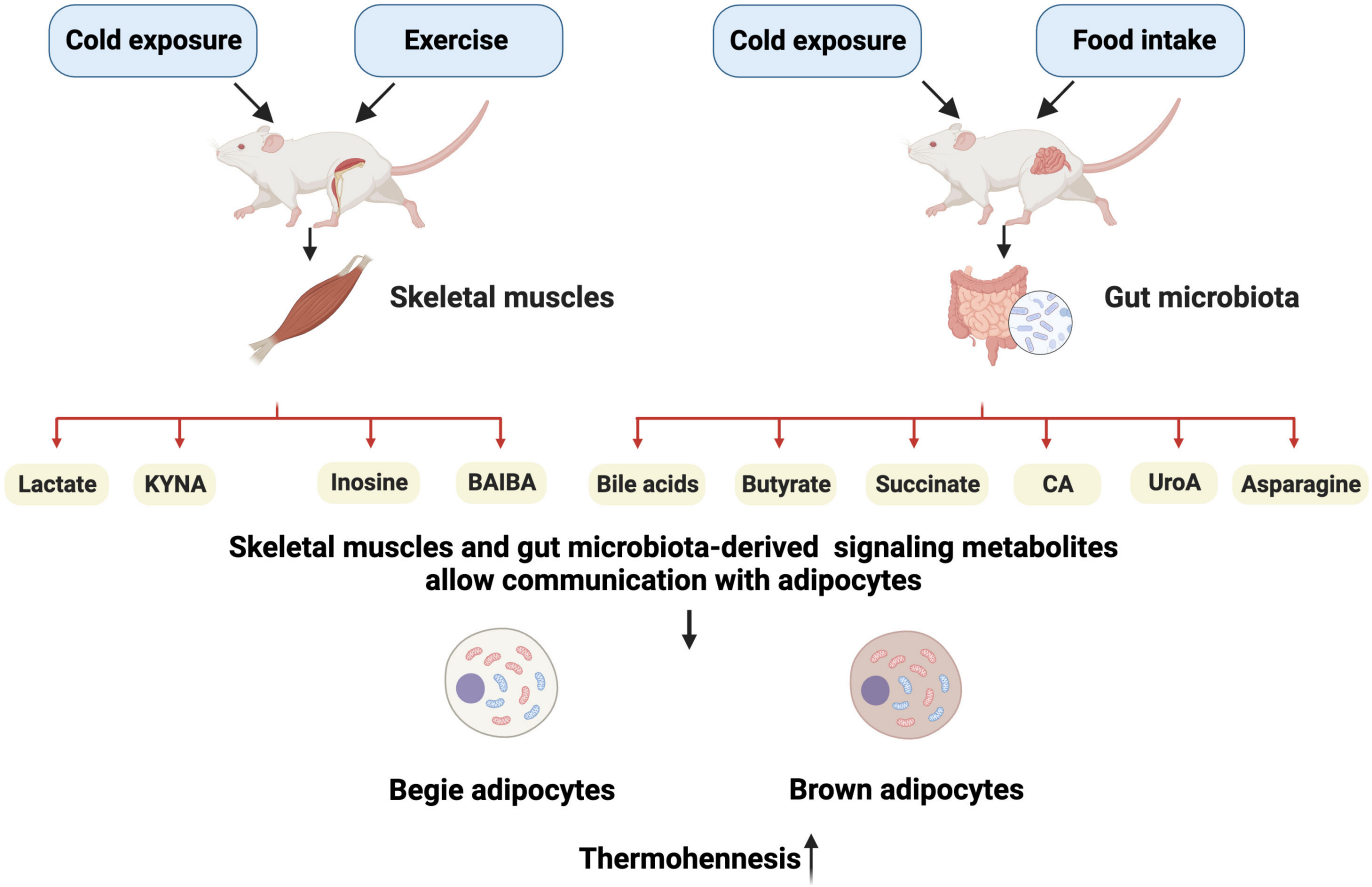
Schematic representation of main metabolites targeting the adipose tissue. Upon stimulation by external factors, skeletal muscles can release lactate, kynurenic acid (KYNA), inosine, and β-aminoisobutyric acid (BAIBA), whereas the gut secretes bile acids (BAs), butyrate, succinate, cinnabarinic acid (CA), urolithin A (UroA), and asparagine. These metabolites can stimulate adipocyte heat generation by acting on specific targets. Created with BioRender.com.

In this review, we focus on the recent findings on skeletal muscles and gut microbiota-derived metabolites that potentially promote adipocyte thermogenesis (**Figure 1**). Some of these metabolites that are released in response to muscle contraction have been reported to mediate the beneficial effects of exercise in thermogenesis, such as lactate, kynurenic acid (KYNA), inosine, and β-aminoisobutyric acid (BAIBA). The gut microbiota can metabolize dietary nutrients into many metabolites, including bile acids (BAs), butyrate, succinate, cinnabarinic acid (CA), urolithin A (UroA), and asparagine. The mechanism by which these metabolites act as signaling molecules to promote adipose tissue thermogenesis is more clearly identified by interacting with membrane receptors and controlling intracellular enzyme activity. Future obesity research will focus on the mechanisms behind the reciprocal exchange of metabolites between adipose tissue and other metabolic organs. Furthermore, a better understanding of how metabolites control adipose tissue activity will enable the identification of novel treatment targets for obesity.

## 2 Skeletal muscle metabolites

**Figure 2:**
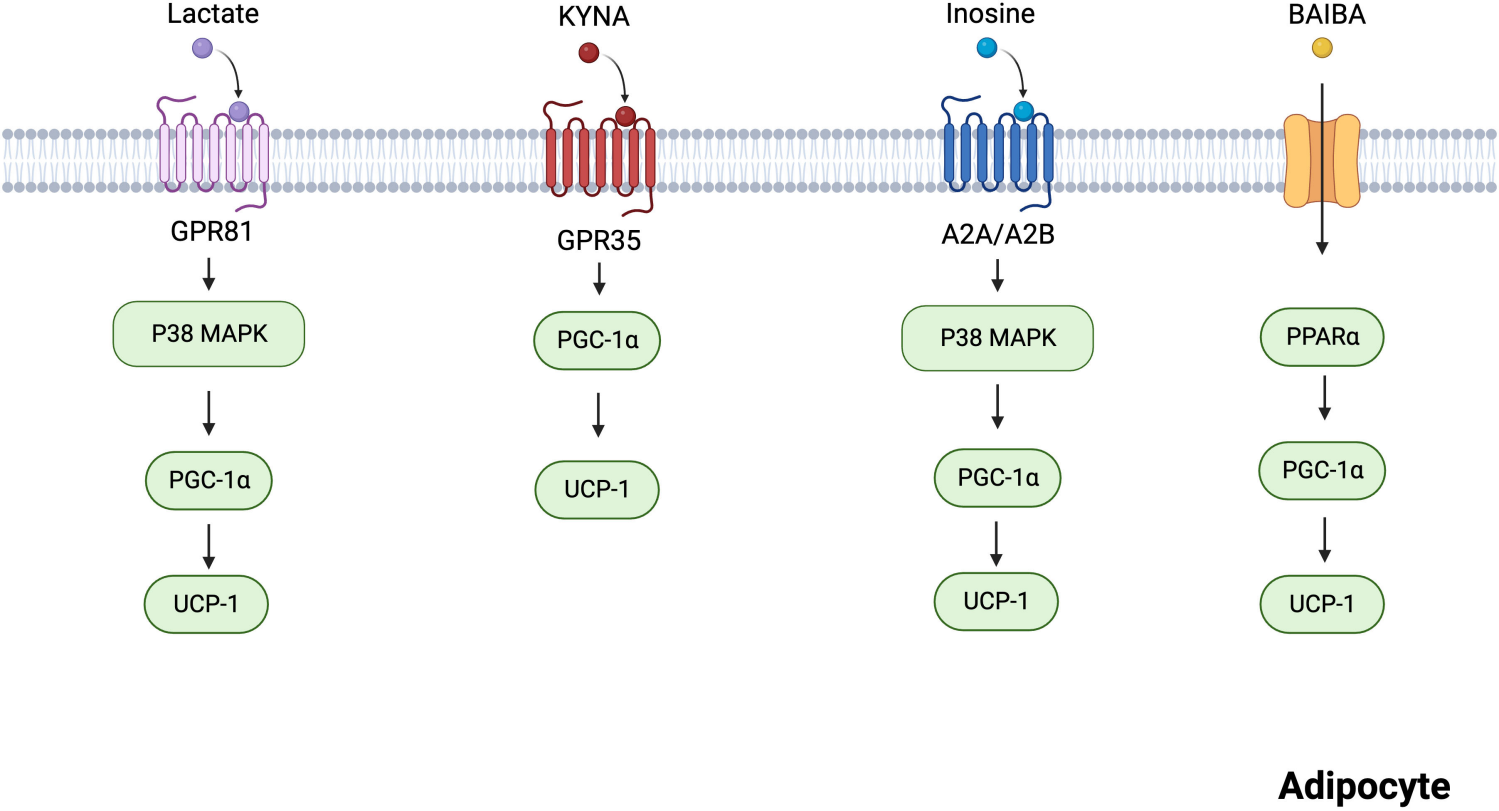
Mechanisms of several skeletal muscles-derived metabolites regulating adipocytes thermogenesis. Lactate acts on GPR81 and activates the p38 MAPK pathway to promote thermogenesis. KYNA promotes the expression of thermogenesis-related genes via GPR35. Inosine promotes the p38 MAPK pathway via A2A/A2B, incresing the expression of thermogenic markers. BAIBA increases the expression of thermogenic genes through PPARα. Created with BioRender.com.

The skeletal muscle is a thermogenic organ that plays a role in maintaining body temperature. Cold conditions trigger the rapid contraction of skeletal muscles, leading to heat production. In recent years, skeletal muscle has been recognized as the main site of shivering thermogenesis in mammals and an endocrine organ. Skeletal muscles produce myokines in response to exercise, allowing crosstalk between muscles and other organs, including the brain, adipose tissue, and gut (29). For example, exercise-mediated lipolytic myokines (interleukin 6, irisin, and leukemia inhibitory factor) stimulate thermogenesis by promoting adipocyte browning (19, 30). Recent research has shown that many metabolites, including lactate, KYNA, inosine, and BAIBA, are produced by skeletal muscles in response to cold exposure and strenuous exercise (31–33). In this section, we focused on these metabolites that can act on specific G protein-coupled receptors (GPCRs) and enzymes to promote BAT activity and WAT browning (**Figure 2**).

### 2.1 Lactate

Lactate is a metabolic byproduct of aerobic glycolysis, and its production is higher than normal during strenuous aerobic exercise owing to the increased oxygen requirement of the muscles (34). Lactate has been previously considered a metabolic waste product, lacking biological function (35). However, it was recently identified as a signaling molecule that regulates lipid metabolism, even under aerobic conditions (36, 37). Studies have shown that fasting plasma lactate levels are higher in obese individuals with metabolic syndrome than in healthy lean individuals (38, 39). Consistent with human findings, obese mice exhibited higher plasma lactate levels than lean mice (40). In contrast, WAT lactate concentration was markedly lowered in obese mice (40), suggesting that WAT utilizes lactate. Yao et al. (40) demonstrated that dietary lactate reduces anomalies in lipid metabolism, improves adipose browning, and increases thermogenesis.

G protein-coupled receptor 81 (GPR81), a specific GPCR for lactate, is primarily expressed in the adipose tissue (41, 42). A study reported that lactate upregulates p38 activation via GPR81 in white adipocytes (40). The p38 mitogen-activated protein kinase (MAPK) mediated adipose browning by activating peroxisome proliferator-activated receptor γ coactivator 1α (PGC-1α) and UCP1 (43). However, GPR81 deficiency significantly attenuated adipose browning and thermogenesis (40). Therefore, GPR81 may be a novel molecular therapeutic target for obesity. Although lactate could not directly activate peroxisome proliferators-activated receptors (PPARγ), it activated PPARγ-dependent browning signaling pathways in white adipocytes (44). In brown adipocytes, the expression of lactate-induced fibroblast growth factor 21 (FGF21) through the activation of the p38-MAPK pathway promoted adipocyte browning and thermogenesis (45, 46).

### 2.2 Kynurenic acid

KYNA is a significant bioactive product in tryptophan metabolism (47). Given that KYNA has neuroprotective properties, it has been the subject of intensive research over the past few decades (48). Growing evidence shows that KYNA exerts protective effects against metabolic diseases, such as obesity and non-alcoholic fatty liver disease (49). In a pilot clinical trial, KYNA serum levels were lower in obese individuals than in healthy individuals (49). Furthermore, endurance exercise increased plasma KYNA levels via KYNA synthesis in skeletal muscle (50, 51).

KYNA plays an important role in adipose tissue energy metabolism. KYNA-treated 3T3-L1 adipocytes exhibited reduced lipogenesis inflammatory response and insulin resistance (52). In mice, KYNA prevented high-fat diet (HFD)-induced body weight gain and reduced serum triglyceride levels (53, 54). Agudelo et al. also demonstrated that KYNA increases energy utilization by activating GPR35, which stimulates lipid metabolism and thermogenic and anti-inflammatory gene expression in adipose tissues (53). Based on these findings, KYNA is an important signaling molecule involved in energy homeostasis.

### 2.3 Inosine

Inosine is a crucial secondary metabolite in purine metabolism (55). In a longitudinal cohort study, physical activity increased plasma inosine levels (31). Equilibration nucleoside transporter 1 (ENT1), a member of the SLC29 family, is an inosine transporter that regulates extracellular inosine concentrations (56). Niemann et al. established a relationship between high levels of mutant ENT1 and a low body mass index (BMI) (57). In addition, the adipose tissue-specific knockout of ENT1 in mice fed an HFD resulted in reduced lipid accumulation and increased thermogenesis (57). Similarly, inosine-treated mice fed an HFD gained significantly less weight and showed elevated expression of thermogenic markers, including UCP1 and PGC-1α (57). Inosine activates four adenosine receptors, including A1, A2A, A2B, and A3 (58), among which A2A and A2B are highly expressed in BAT (59). Inosine activates A2A/A2B and stimulates thermogenesis via the cyclic adenosine monophosphate (cAMP)-p38 pathway (57). In addition, pharmacological stimulation with A2A contributes to the browning of white adipocytes (59). These findings indicate that inosine is a potential regulator of energy homeostasis via A2A and A2B.

### 2.4 β-aminoisobutyric acid

BAIBA is a metabolite of valine and is mainly produced by muscle contraction during exercise (33, 60). Under *in vivo* and *in vitro* conditions, BAIBA promoted WAT browning (44, 61). In white adipocytes, BAIBA upregulated the expression of thermogenic genes, including UCP-1, PGC-1α, and cytochrome c (62). However, BAIBA did not increase the expression of thermogenic genes in PPARα null mice (62). Therefore, BAIBA promotes beige fat formation through PPARα. BAIBA also induced adipocytes to secrete leptin, which promoted white adipocyte browning by inhibiting the Hh signaling pathway (63, 64). However, the mechanisms underlying how BAIBA promotes leptin secretion from adipocytes remain unclear and require further investigation.

Adipose tissue browning may improve plasma lipid profiles and blood glucose levels (65). In a large human cohort study, plasma BAIBA levels were inversely correlated with metabolic risk factors, such as BMI, triglycerides, and fasting glucose (62). Similarly, HFD-fed mice treated with BAIBA exhibited reduced weight gain and improved insulin resistance (66). Furthermore, BAIBA-treated 3T3-L1 cells showed enhanced browning phenotype, lipid accumulation suppression, and insulin resistance mitigation (67, 68). These findings suggest that BAIBA is a potential therapeutic option for the treatment of obesity and its associated metabolic diseases.

## 3 Gut microbiome metabolites

The diverse microbial community in the gut, known as the gut microbiota, regulates appetite, energy absorption, and lipid and glucose metabolism (69). Accumulating evidence suggests a direct causal relationship between gut microbiota and obesity. In one study, germ-free mice did not become obese even when fed HFD, compared with mice with microbiota. When the gut microbiota of obese mice were transplanted into germ-free mice, the body weight of the transplanted mice significantly exceeded that of the control group that was transplanted with healthy mouse microbiota after 2 weeks; this suggests that the obesity phenotype can be transferred between different individuals through microbiota (70). Recently, a link between gut microbiota and obesity has been observed in humans. Recent studies have confirmed a strong association between the abundance of certain gut bacteria and obesity, as indicated by abnormal body weight or BMI. For example, the abundance of Akkermansia muciniphila, a bacterium belonging to the Verrucomicrobia phylum, exhibited a significant negative correlation with fasting blood glucose levels, waist-to-hip ratio, and subcutaneous fat cell diameter (71). A high abundance of Bacteroides and high organic acid contents were observed in obese people in Denmark, while a decrease in the abundance of butyric-producing bacteria was observed in non-obese people, suggesting that microbial metabolites may also play a role in obesity (72). Further evidence shows that the lack of HIF-2α specifically in the gut resulted in an imbalance between Bacteroides vulgatus and Ruminococcus torques. This imbalance significantly increased the levels of taurine-binding cholic acid and deoxycholic acid and activated TGR5 in WAT, which further upregulated the expressions of UCP1 and CKMT2, thereby promoting body thermogenesis. Moreover, after antibiotic clearance of intestinal microbes, the loss of intestinal HIF-2α no longer affected heat production in WAT (73). This suggests that gut microbiota can improve obesity by promoting fat thermogenesis through the action of metabolites.

Microbial metabolites, which serve as the bridge between diet (microbiota) and obesity, are of great value for understanding the development of obesity. Previous research suggests that microbial metabolites may be effective targets for controlling obesity (74). Metabolites are “signaling molecules” that are released into the extracellular environment and can mediate these effects (75). The metabolites secreted by the gut are mostly derived from two sources. First, the brain controls intestinal function through nerve conduction in response to external environmental stimuli (e.g., cold exposure and exercise), which affects the secretion of metabolic products by the gut microbiota (76, 77). Second, the gut microbiota transforms food molecules into metabolites (78). Thus, metabolites serve as informational mediators of the host–microbiome crosstalk.

**Figure 3:**
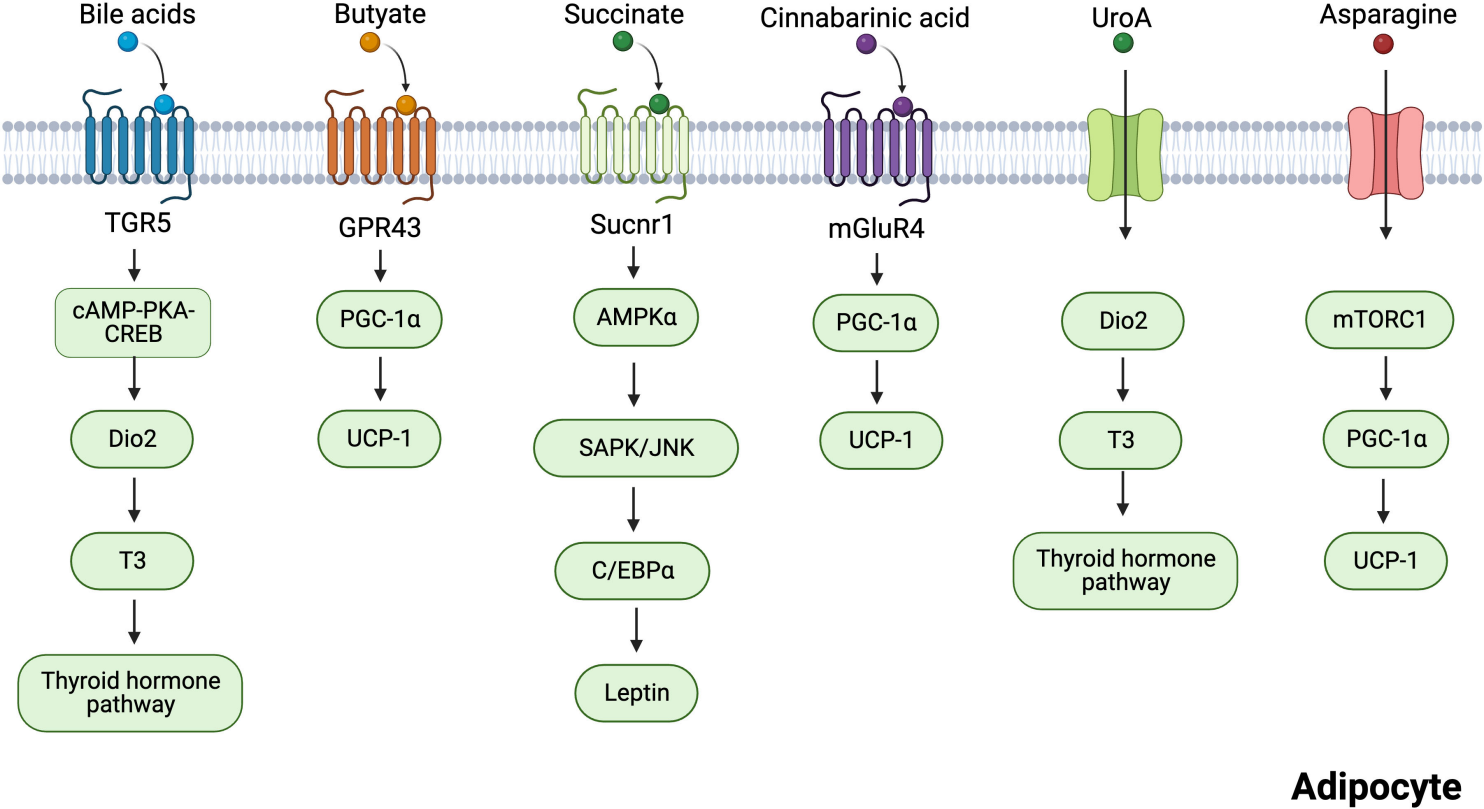
Mechanisms of several gut microbiota-derived metabolites regulating adipocytes thermogenesis. Bile acids induce the transcription of cAMP-PKA-CREB-induced Dio2 via the TGR5 receptor. Dio2 increased thermogenesis in adipose tissues by promoting the conversion of inert thyroxine T4 to T3. Butyrate promotes the expression of thermogenesis-related genes through GPR43. Succinate activates sucnr1 receptors to release leptin in an AMPK/JNK-C/EBPα-dependent manner, and leptin can stimulate PGC-1α and PPARα expression to promote thermogenesis. Cinnabarinic acid promotes the expression of thermogenesis-related genes through mGluR4. UroA acts on Dio2 and promotes the conversion of T3 to activate the thyroid hormone pathway. Asparagine activates mTORC1 signaling pathway, which increases the expression of PGC-1α, thus enhancing thermogenesis. Created with BioRender.com.

Six intestinal metabolites, including BAs, butyric acid, succinate, CA, UroA, and asparagine (**Figure 3**), are discussed in the subsequent section. Their roles in regulating adipose tissue thermogenesis are explored, and their targets in adipocytes are summarized.

### 3.1 Bile acids

BAs are cholesterol derivatives and major regulators of lipid metabolism and EE in host cells (79). There are two pathways for synthesizing BAs: classical and alternative pathways. The classical pathway is controlled by CYP8B1 and mainly synthesizes 12-position hydroxy (12-OH) BAs. The alternative pathway is mainly controlled by CYP7B1 and synthesizes non-12-OH BAs (80). In a human cohort study, unhealthy individuals with high BMI had lower levels of non-12-OH BAs (81). The same study revealed that mice with slow weight gain exhibited higher levels of non-12-OH BAs than obesity-prone mice and that these mice had fewer metabolic disturbances (81). Overall, these findings suggest that non-12-OH BAs are closely associated with metabolic states in obesity.

Cold exposure triggers new metabolic mechanisms and increases EE. In a particular study, cold exposure was found to promote the conversion of cholesterol to BAs via alternative pathways and increase the production of non-12-OH BAs (76). cyp7b1-/- mice showed significant downregulation of UCP-1 expression in brown fat (76), whereas cyp8b1 knockout mice exhibited resistance to HFD-induced obesity (82). A recent study demonstrated that supplementation with non-12-OH BAs promotes thermogenesis and improves weight regain in mice resuming food intake after a calorie-restricted diet (83). These findings suggest that non-12-OH BAs can promote EE and improve obesity.

BA signaling is mainly mediated by the nuclear farnesoid X receptor (FXR) and G-protein-coupled bile acid receptor 5 (TGR5) (84). TGR5 signaling confers the numerous advantageous effects of BAs, including the prevention of fatty degeneration, alterations in blood glucose levels, and promotion of energy homeostasis (85–87). Moreover, non-12OH BAs have been demonstrated to improve the energy metabolism of white and brown fat through TGR5-mediated activation of BAT and upregulation of UCP1 expression (88). In WAT and BAT, BAs induced the transcription of cAMP-protein kinase A-cAMP response element-binding protein (cAMP-PKA-CREB)-induced deiodinase 2 (Dio2) via the TGR5 receptor (89). Dio2 increased thermogenesis in adipose tissues by promoting the conversion of inert thyroxine T4 to T3 (the active form of thyroid hormone) (89). Administration of TGR5 with the agonist INT-777 increased the number of mitochondria in BAT. However, this was not observed in TGR5-knockout animals (86). These findings suggest that TGR5 is necessary for thermogenesis.

### 3.2 Butyrate

Short-chain fatty acids (SCFAs) are the most abundant metabolites during microbial fermentation of dietary fiber. Acetate, propionate, and butyrate account for over 95% of SCFAs and are produced by specific bacteria. Acetate is primarily produced by Bacteroides, Bifidobacterium, Streptococcus, Streptococcus peptica, Clostridium, and Rumex coccus. Butyrate is produced by Bacteroides, Eubacterium, and Clostridium (90). Propionate is produced by Clostridium and Bacteroides (91). Furthermore, accumulating evidence suggests that butyrate is a major regulator of tissue function in SCFAs, which affects systemic energy metabolism (92).

A stable isotope study showed that butyrate production was negatively correlated with BMI (93). Similarly, the microbiota of obese mice produced lower levels of butyrate than that of lean mice (94). Studies on the effects of butyrate on mice have shown that long-term supplementation with 5% sodium butyrate prevents HFD-induced weight gain and reduces fat mass (95). Moreover, butyrate reduced HFD-induced hyperglycemia and hyperinsulinemia (96, 97). These results suggest that butyrate reduces obesity and obesity-related metabolic disorders.

Cold exposure is an important environmental factor that promotes thermogenesis and increases whole-body EE. A study showed that cold exposure directly increases butyrate concentrations in the cecum, suggesting that butyrate plays an important role in maintaining body temperature (77). An important mechanism by which butyrate increases EE is by promoting thermogenesis in adipocytes. The addition of butyrate increased BAT and WAT thermogenesis (98). Although microbiota depletion decreased thermogenesis, this effect was reversed by butyrate supplementation (99), indicating that butyrate is an important mediator of lipid thermogenesis.

Butyrate acts as a signaling molecule by activating GPR43, GPR41, and GPR109a receptors (100). Many studies have shown that GPR43 is expressed in human and mouse WAT and the mouse adipocyte cell 3T3L1 (101). GPR41 is also expressed in human adipose tissue, but to a lesser extent than GPR43 (102). Brown et al. reported that GPR43 expression is higher in the WAT of HFD-fed obese mice than in mice fed a normal diet (101). Moreover, mice with low GPR43 expression gained weight even when fed a standard diet (90). PGC-1α mRNA expression in BAT under butyrate treatment was positively correlated with GPR43 levels (90). These results suggest that butyrate regulates BAT thermogenesis through GPR43. Leptin suppresses appetite and promotes thermogenesis and fat burning in the body (103). Butyrate directly stimulated adipocyte leptin production by activating the GPR41 and GPR43 signaling pathways (104). A recent study found that butyric acid also acts as an epigenetic regulator, regulating thermogenic gene expression in BAT and subcutaneous WAT (scWAT) by activating lysine-specific histone demethylase 1 (LSD1) (105).

### 3.3 Succinate

Succinate is an important metabolite in host–bacterial interaction, an intermediary of the host tricarboxylic acid cycle, and a fermentation product of intestinal flora (106, 107). In a large human cross-sectional study, negative correlations were observed between plasma succinate levels and total and visceral obesity (108). Succinate is associated with energy metabolism in adipose tissue. Succinate is an intracellular signaling molecule that regulates the physiological function of BAT by acting as a thermogenic agent (107). Based on stable isotope tracers, blood succinic acid levels in mice increased when cold stimulated, and it was found that these succinic acids preferentially accumulated in brown fat. This means that succinic acid is involved in fatty tissue (107). More importantly, succinate has been reported to stimulate thermogenesis in the brown adipocytes and BAT of mice via the succinate dehydrogenase-mediated production of reactive oxygen species (107). Succinate is sensed extracellularly by succinate receptor 1 (SUCNR1) (109), which is highly abundant in WAT and mediates the antilipolytic activity of succinate (110, 111). Furthermore, succinate increased the browning of adipose tissue in Crohn’s disease (60). A positive correlation existed between VAT-derived stem cell SUCNR1 mRNA and circulating succinate levels (112). Leptin can stimulate PGC-1α and PPARα expression to promote thermogenesis (113, 114). A recent study showed that succinate signaling modulates energy homeostasis by regulating adipocyte leptin production (115). SUCNR1 activation controlled leptin expression in an AMPK/CCAAT/enhancer-binding protein alpha/c-Jun N-terminal kinase (AMPK/JNK-C/EBPα)-dependent manner (115). Adipocyte-specific Sucnr1 knockout (Ad-Sucnr1 KO) mice displayed reduced levels of subcutaneous and visceral WAT (115). SUCNR1 activation promotes an anti-inflammatory phenotype in macrophages, whereas myeloid-specific SUCNR1 deficiency hinders adipose tissue browning (109). Overall, the mechanisms involved are unclear, and therefore, further studies are required.

### 3.4 Cinnabarinic acid

CA is a tryptophan metabolite (116). Exogenous and endogenous amino acids obtained from food *in vitro* and the breakdown of tissue proteins, respectively, serve as the primary sources of tryptophan in animals (117). Tea contains a small amount of tryptophan, which aids digestion and metabolism, thereby increasing CA levels in plasma (117, 118). Earlier investigations indicated that CA possesses anti-inflammatory and antioxidant activity and protects hepatocytes (119, 120). However, recent research has revealed that CA is a crucial metabolite in the weight reduction activity of Pu-erh tea (117). CA-treated mice were found to have significantly higher levels of thermogenic proteins in BAT and lower levels of WAT deposition in the epididymis than untreated mice (117). Metabolic glutamate receptor 4 (mGluR4) is the target of CA (121). CA treatment increases mGluR4 expression in WAT and BAT, which promotes lipolysis and thermogenesis (117).

### 3.5 Urolithin A

UroA is an intestinal metabolite produced by foods containing ellagic acid, such as pomegranate, berries, and walnuts (122). A relationship exists between the type of urolithin production and specific intestinal bacteria, with Gordonibacter producing mainly UroA (123). The differences in ellagic acid metabolism between healthy overweight-obese individuals and normal-weight individuals were analyzed, and UroA levels were found to be higher in the normal-weight group than in the overweight-obese group (124). Additionally, correlation analysis revealed that UroA was positively correlated with apolipoprotein A-I and intermediate-high-density lipoprotein-cholesterol (125). These results suggested that UroA has potential anti-obesity effects. Emerging evidence suggests that UroA regulates energy metabolism in various cells. UroA inhibited the expression of genes related to adipogenesis and lipid accumulation in 3T3-L1 adipocytes (126). Supplementation with UroA significantly enhanced healthy metabolism in HFD mice, for example, by reducing obesity and hyperglycemia (127). UroA improves obesity by increasing EE. Mechanistically, UroA increased the conversion of inactive T4 to active T3 by triggering Dio2 (127). Activation of the thyroid hormone pathway enhanced BAT thermogenesis and induced WAT browning (127). In several clinical trials, UroA improved mitochondrial activity and muscle strength while being safe and well-tolerated (128–130). Therefore, UroA may be considered as a potential therapy for alleviating obesity.

### 3.6 Asparagine

Asparagine is a nonessential amino acid, most of which is synthesized by the body itself, but the gut flora is also thought to be a source of asparagine synthesis (131). Many foods, such as dairy and meat, contain high levels of asparagine (132). These foods are absorbed by intestinal microbes, thereby increasing asparagine levels in the body (132). Clinical metabolomic investigations have revealed a negative correlation between plasma asparagine levels and metabolic syndrome (133, 134). Asparagine significantly improved the ability of mice to maintain body temperature during cold exposure and prevented weight gain (135). However, metabolic disturbances were observed when asparaginase was administered to remove circulating asparagine (136, 137). Mechanistically, asparagine activated the mechanistic target of the rapamycin complex 1 (mTORC1) signaling pathway, which increased the expression of PGC-1α, thus enhancing thermogenesis in BAT while also promoting beige coloring in WAT (135). Overall, asparagine functions as a signaling molecule that promotes thermogenesis via mTORC1.

## 4 Conclusions and perspective

In this review, we outline the roles of metabolic products secreted by the skeletal muscle and gut as signaling molecules that promote heat production in adipocytes: (i) Skeletal muscles can release lactate, kynurenic acid, inosine, and β-aminoisobutyric acid during exercise. (ii) The gut secretes bile acids, butyrate, succinate, cinnabarinic acid, urolithin A, and asparagine stimulated by diet and cold exposure. (iii) These metabolites can act as signaling molecules that mediate thermogenesis by binding to receptors and enzymes. We emphasize the essential roles of circulating metabolites in the total body energy balance and their functions as significant mediators of interorgan communication and metabolic adaptability of the entire organism.

Medications that promote the generation of fat heat, such as capsaicin and 3-AR agonists, have been developed (138–140); However, their uses are limited by negative effects. In the future, it is necessary to develop drugs with lower toxicity and improved efficacy. In this review, we find that many metabolites can activate GPCRs, which are relatively ‘easy’ drug targets (141, 142), and metabolite-GPCRs constitute a promising and as yet underutilized pharmacotherapeutic. These metabolites could become important treatment options in the management of cellular metabolism, and more importantly, in the management of metabolic disorders. However, there are uncertainties and limitations due to interspecific differences (humans and mice) as well as individual variations in clinical studies. Consequently, long-term conversion research and clinical trials are required to evaluate the dosage, thermogenic effects, and other aspects of metabolic supplements.

## Author contributions

YT: Writing – original draft, Writing – review & editing. Y-DW: Writing – original draft. Y-YW: Investigation, Writing – review & editing. Z-ZL: Data curation, Writing – review & editing. X-HX: Supervision, Writing – review & editing.
